# Chloral in Tetanus

**Published:** 1871-05

**Authors:** 


CHLORAL IN TETANUS.—The journals in Europe and America are reporting a considerable number of cases of tetanus, mostly idiopathic, treated by chloral, some of which have recovered, whilst the great majority prove fatal. An interesting case will be found in this journal, which terminated favorably, reported by Dr. Cluness. The *Australian Medical Journal*, for November last, contains the report of a similar case, in which the symptoms were much relieved at first by the remedy. The patient died, however, in five days from the attack. The quantity of chloral administered during the four days of treatment was 560 grains.—*Pacific Medical Journal.*

